# Supplementary material for: An Investigation of the Protein Quality and Temporal Pattern of Peripheral Blood Aminoacidemia following Ingestion of 0.33 g·kg−1 Body Mass Protein Isolates of Whey, Pea, and Fava Bean in Healthy, Young Adult Men
Source: Nutrients. 2023 Sep 29;15(19):4211. doi: 10.3390/nu15194211 (PMC10574361; doi:10.3390/nu15194211)

Supplementary Figure S1. CONSORT flow diagram of participant enrolment, allocation, follow-up, and analysis.

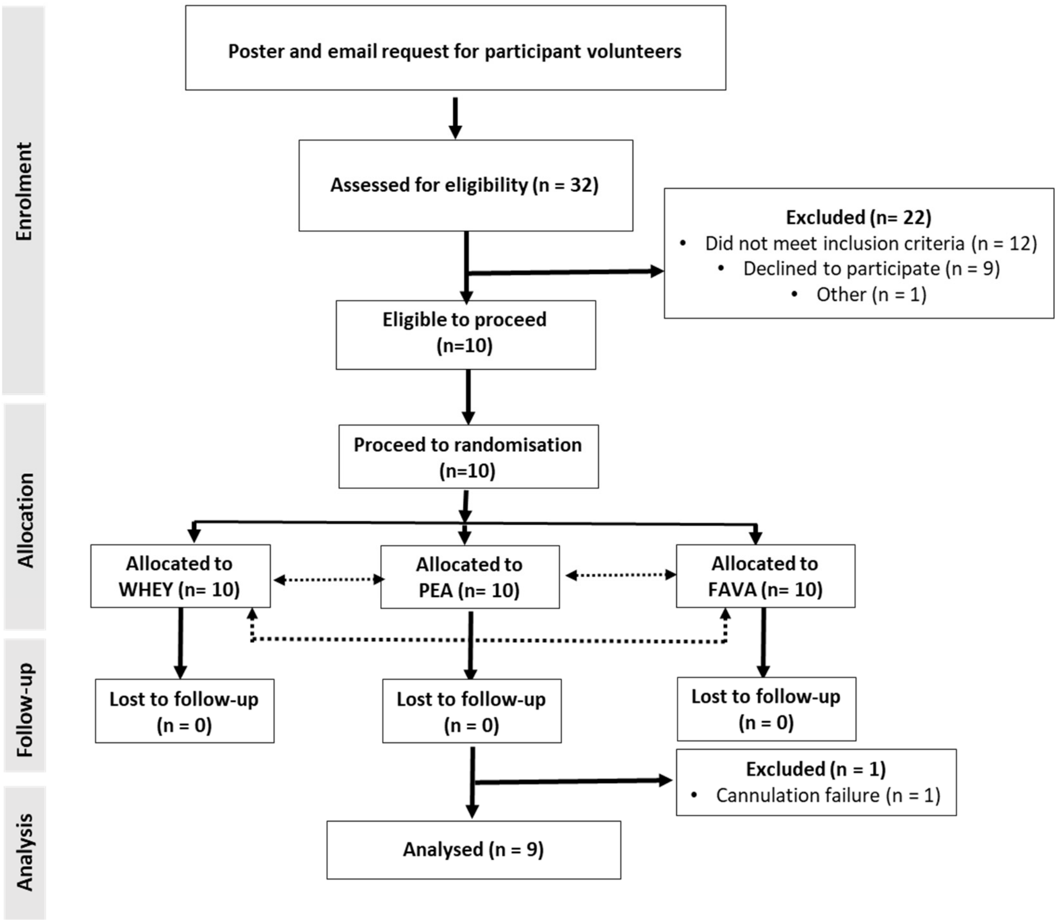

Supplement: Supplementary file 1 [file nutrients-15-04211-s001.zip › Supplementary Figure S1.pdf]
