# Supplementary material for: An Investigation of the Protein Quality and Temporal Pattern of Peripheral Blood Aminoacidemia following Ingestion of 0.33 g·kg−1 Body Mass Protein Isolates of Whey, Pea, and Fava Bean in Healthy, Young Adult Men
Source: Nutrients. 2023 Sep 29;15(19):4211. doi: 10.3390/nu15194211 (PMC10574361; doi:10.3390/nu15194211)
Supplement: Supplementary file 1 [file nutrients-15-04211-s001.zip › Supplementary Table S1.pdf]

Supplementary Table S1. Compositional analysis of the protein isolates.

	WHEY[1]	PEA[2]	FAVA[3]	WHEY	PEA	FAVA
Moisture, 105°C (%)	6%	7%	4%	6%	7%	4%
%N in amino acids				11.35%	10.97%	9.83%
NPCF[4]	6.25	6.25	6.25	6.75	6.13	6.07
Protein (Dry basis) (%)	77%	83%	78%	81%	81%	75%
Ash, 450°C (g)	5.8	3.87	6.6	5.8	3.87	6.6
Total carbohydrate (g)	1.3	2	3.1	1.3	2	3.1
	($g \cdot 100^{-1}$ product)			($g \cdot 100^{-1}$ protein)[4]		
Histidine	1.61	2.19	1.91	1.98	2.71	2.54
Isoleucine	5.03	3.8	3	6.20	4.69	3.98
Leucine	9.35	6.53	5.66	11.52	8.07	7.52
Lysine	8.1	6.03	4.81	9.98	7.45	6.39
Methionine + cysteine	3.74	2.55	1.29	4.61	3.15	1.71
Methionine	1.61	0.91	0.524	1.98	1.12	0.70
Cysteine	2.13	1.64	0.77	2.62	2.03	1.02
Phenyalanine + tyrosine	5.55	6.66	5.87	6.84	8.23	7.79
Phenylalanine	2.85	4.39	3.35	3.51	5.42	4.45
Tyrosine	2.7	2.27	2.52	3.33	2.80	3.35
Threonine	6.44	2.81	2.56	7.94	3.47	3.40
Tryptophan	1.24	0.71	0.61	1.53	0.88	0.82
Valine	5.09	4.22	3.37	6.27	5.21	4.47
Σ Indispensable (g)	46	36	29	57	44	39
% Indispensable	52%	46%	42%	52%	46%	42%
Alanine	4.26	3.44	2.96	5.25	4.25	3.93
Arginine	2.39	6.69	6.52	2.95	8.27	8.66
Aspartic acid	9.51	8.57	8.35	11.72	10.59	11.09
Glutamic acid	15.22	13.58	12.4	18.76	16.78	16.46
Glycine	1.66	2.92	2.94	2.05	3.61	3.90
Proline	5.04	3.33	3.24	6.21	4.11	4.30
Serine	4.72	3.91	3.76	5.82	4.83	4.99
Σ Dispensable (g)	43	42	40	53	52	53
% Dispensable	48%	54%	58%	48%	54%	58%
Total AA (g)	89	78	69	110	96	92

[1] WHEY: isolated from liquid whey from cheesemaking, pasteurised, membrane filtered and spray dried.

[2] PEA: isolated from 100% non-GMO Canadian peas by natural biochemical separation.

[3] FAVA: liquid isolate from milled flour, pasteurised, microfiltered and spray dried.

[4] NPCF factor calculated from the amino acid profile (FAO N-Factor calculator, www.fao.org)
