# Supplementary material for: An Investigation of the Protein Quality and Temporal Pattern of Peripheral Blood Aminoacidemia following Ingestion of 0.33 g·kg−1 Body Mass Protein Isolates of Whey, Pea, and Fava Bean in Healthy, Young Adult Men
Source: Nutrients. 2023 Sep 29;15(19):4211. doi: 10.3390/nu15194211 (PMC10574361; doi:10.3390/nu15194211)
Supplement: Supplementary file 1 [file nutrients-15-04211-s001.zip › Supplementary Table S2.pdf]

Supplementary Table S2: Estimate of the dietary indispensable amino acid score (DIAA) for adults for the whey, pea, and fava protein isolates based on FAO, 2013.

		WHEY				PEA				FAVA			
Amino acid	IAA reference pattern	IAA in CP	TID[18]	TID IAA	DIAA reference ratio	IAA in CP	TID[19]	TID IAA	DIAA reference ratio	IAA in CP	TID[20]	TID IAA	DIAA reference ratio
	(mg/g)	(mg/g)		(mg/g)		(mg/g)		(mg/g)		(mg/g)		(mg/g)	
Histidine	16	21	1.00	21	1.31	27	0.95	25	1.58	25	0.70	17	1.08
Isoleucine	30	66	0.98	64	2.14	46	0.91	42	1.40	39	0.82	32	1.06
Leucine	61	122	0.99	121	1.98	79	0.92	73	1.19	73	0.79	58	0.95
Lysine	48	106	0.98	103	2.16	73	0.96	70	1.46	62	0.73	46	0.95
Methionine + cysteine (SAA)	**23**	49	0.98	48	2.08	31	0.83	26	1.11	17	0.76	13	**0.55**
Methionine	*16*	21	0.98	21	**1.29**	11	0.90	10	**0.62**	7			
Cysteine	*6*	28	0.98	27	4.54	20	0.75	15	2.48	10			
Phenylalanine + tyrosine (AAA)	41	72	0.99	71	1.74	81	0.93	75	1.82	76	0.64	49	1.19
Phenylalanine		37	0.98	36		53	0.92	49		43			
Tyrosine		35	0.99	35		28	0.93	26		33			
Threonine	25	84	0.94	79	3.16	34	0.88	30	1.20	33	0.69	23	0.91
Tryptophan	6.6	16	1.00	16	2.45	9	0.89	8	1.16	8	0.57	4	0.68
Valine	40	66	0.97	64	1.61	51	0.89	46	1.14	43	0.76	33	0.83
		**DIAAS for WHEY (%) 129 (Met)**				**DIAAS for PEA (%) 62 (Met)**				**DIAAS for FAVA (%) 55 (SAA)**			

IAA: Indispensable amino acid
CP: Core protein
TID: True ileal indispensable amino acid digestibility coefficient (predicted human values obtained from pig data (source reference in superscript)
SAA: Sulphur amino acids
